# Editorial

**Published:** 1902-12-15

**Authors:** 


### EDITORIAL.

The committee having in charge the matter of getting dental surgeons enlisted as commissioned officers in the navy has again taken up its work in an earnest and aggressive manner. The need of dental service in the navy is recognized by all the officials, and all are desirous of providing this service. The expense involved never seems to have been raised, but the naval authorities do not wish to increase the commissioned officers by adding another class. The reasons given are entirely sentimental and ought not to be considered ; but the jealousies existing among commissioned officers in the government service are so universal that rules of action are generally based upon them. It therefore will be a difficult matter to get the naval committees of Congress to report a bill recommending the enactment of a law providing commissioned officers of dental surgeons. The bill proposing *commissioned status* for dental surgeons for the navy is now in the hands of the committee of which Hon. Jos. E. Watson, of Indiana, is chairman, and A. G. Dayton, of West Virginia, R. W. Taylor, of Ohio, and Chas. K. Wheeler, of Kentucky, are members. If any of our readers can furnish these gentlemen or any one of them with such enlightenment as shall cause them to give this subject the favorable consideration it demands from our standpoint, it is his duty to use all his powers to the end that this matter may be put on a professional basis. We don't want yeoman dentists in the navy, neither do we want contract dental surgeons, but the dignity of our calling demands service on a commissioned basis.

In our October issue, page 508, we printed an abstract from the proceedings of the Kansas State Dental Association, and credited it to the Missouri State Dental Society. We also failed to credit the reprint to the *Western Dental Journal*, all of which was wholly an oversight. We can only say that the article was good enough to emenate from Missouri, and we are not ashamed to have it appear as original matter in the Register.

Dr. L. G. Noel, president of the National Dental Association, was married October 30th, 1902, at Nashville, Tenn., to Miss Augusta Jonnard. The doctor's many friends throughout the country will wish him all the blessings and happiness that come with the completed home life.

Dr. W. A. Price, has invented a new cataphoric apparatus which will soon be put on the market, and which it is confidently expected will make this method of relieving pain in excavating dentine more certain and practicable. It's principal features are a mil-ammeter which is one hundred and twenty-eight times more sensitive than the chloride of silver instrument. The controller has thirty-six hundred steps as compared with one hundred and thirteen in the usual controllers. This perfect control makes its application definite and entirely painless.

